# Bis(3-chloro­benzoato-κ^2^ *O*,*O*′)bis­(nicotinamide-κ*N*)copper(II)

**DOI:** 10.1107/S1600536813014694

**Published:** 2013-06-08

**Authors:** Nihat Bozkurt, Nefise Dilek, Nagihan Çaylak Delibaş, Hacali Necefoğlu, Tuncer Hökelek

**Affiliations:** a Kafkas University, Department of Chemistry, 63100 Kars, Turkey; b Aksaray University, Department of Physics, 68100, Aksaray, Turkey; c Department of Physics, Sakarya University, 54187 Esentepe, Sakarya, Turkey; d Hacettepe University, Department of Physics, 06800 Beytepe, Ankara, Turkey

## Abstract

The mol­ecule of the title Cu^II^ complex, [Cu(C_7_H_4_ClO_2_)_2_(C_6_H_6_N_2_O)_2_], contains two 3-chloro­benzoate (CB) and two nicotinamide (NA) ligands; the CB act as bidentate ligands, while the NA are monodentate ligands. The resulting CuN_2_O_4_ coordination polyhedron is a considerably distorted octahedron. The dihedral angles between the carboxyl­ate groups and the adjacent benzene rings are 17.92 (12) and 24.69 (16)°, while the two benzene rings and the two pyridine rings are oriented at dihedral angles of 52.20 (8) and 1.56 (6)°. In the crystal, N—H⋯N and C—H⋯O hydrogen bonds link the mol­ecules into a three–dimensional network. The π–π contact between the benzene rings [centroid–centroid distance = 3.982 (2) Å] may further stabilize the crystal structure.

## Related literature

For niacin, see: Krishnamachari (1974 ▶). For the nicotinic acid derivative *N*,*N*-di­ethyl­nicotinamide, see: Bigoli *et al.* (1972 ▶). For related structures, see: Greenaway *et al.* (1984 ▶); Hökelek & Necefoğlu (1996 ▶); Hökelek *et al.* (1996 ▶); Hökelek, Dal *et al.* (2009 ▶); Hökelek, Yılmaz *et al.* (2009 ▶); Necefoğlu *et al.* (2011 ▶); Sertçelik *et al.* (2013 ▶).
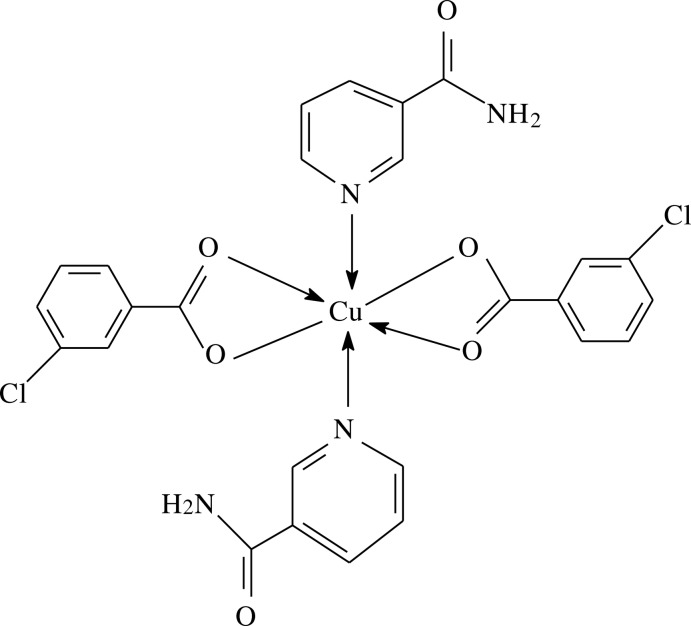


## Experimental

### 

#### Crystal data

[Cu(C_7_H_4_ClO_2_)_2_(C_6_H_6_N_2_O)_2_]
*M* *_r_* = 618.91
Triclinic, 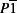

*a* = 9.6614 (2) Å
*b* = 12.5429 (3) Å
*c* = 12.8728 (3) Å
α = 61.598 (2)°
β = 87.386 (3)°
γ = 77.115 (3)°
*V* = 1334.30 (6) Å^3^
*Z* = 2
Mo *K*α radiation
μ = 1.07 mm^−1^
*T* = 296 K
0.35 × 0.20 × 0.15 mm

#### Data collection

Bruker SMART BREEZE CCD diffractometer
Absorption correction: multi-scan (*SADABS*; Bruker, 2012 ▶) *T*_min_ = 0.774, *T*_max_ = 0.852
19053 measured reflections
5434 independent reflections
4970 reflections with *I* > 2σ(*I*)
*R*_int_ = 0.022

#### Refinement

*R*[*F* ^2^ > 2σ(*F* ^2^)] = 0.030
*wR*(*F* ^2^) = 0.083
*S* = 1.06
5434 reflections
368 parameters
117 restraints
H atoms treated by a mixture of independent and constrained refinement
Δρ_max_ = 0.43 e Å^−3^
Δρ_min_ = −0.43 e Å^−3^

### 

Data collection: *APEX2* (Bruker, 2012 ▶); cell refinement: *SAINT* (Bruker, 2012 ▶); data reduction: *SAINT*; program(s) used to solve structure: *SHELXS97* (Sheldrick, 2008 ▶); program(s) used to refine structure: *SHELXL97* (Sheldrick, 2008 ▶); molecular graphics: *ORTEP-3* for Windows (Farrugia, 2012 ▶); software used to prepare material for publication: *WinGX* (Farrugia, 2012 ▶) and *PLATON* (Spek, 2009 ▶).

## Supplementary Material

Crystal structure: contains datablock(s) I, global. DOI: 10.1107/S1600536813014694/rk2404sup1.cif

Structure factors: contains datablock(s) I. DOI: 10.1107/S1600536813014694/rk2404Isup2.hkl

Additional supplementary materials:  crystallographic information; 3D view; checkCIF report

## Figures and Tables

**Table 1:** Hydrogen-bond geometry (Å, °)

*D*—H⋯*A*	*D*—H	H⋯*A*	*D*⋯*A*	*D*—H⋯*A*
N2—H2*A*⋯O2^i^	0.80 (2)	2.12 (2)	2.896 (2)	164 (2)
N2—H2*B*⋯O6^ii^	0.84 (3)	2.02 (3)	2.790 (2)	153 (2)
N4—H4*A*⋯O5^i^	0.83 (3)	2.01 (3)	2.817 (2)	164 (2)
N4—H4*B*⋯O4^ii^	0.81 (2)	2.05 (2)	2.836 (2)	162 (3)
C19—H19⋯O1^iii^	0.93	2.45	3.100 (2)	127
C21—H21⋯O5^i^	0.93	2.56	3.416 (2)	154
C24—H24⋯O3^iv^	0.93	2.59	3.475 (3)	158

Symmetry codes: (i) 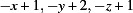; (ii) 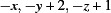; (iii) 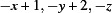; (iv) 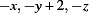.

**Fig. 1.**
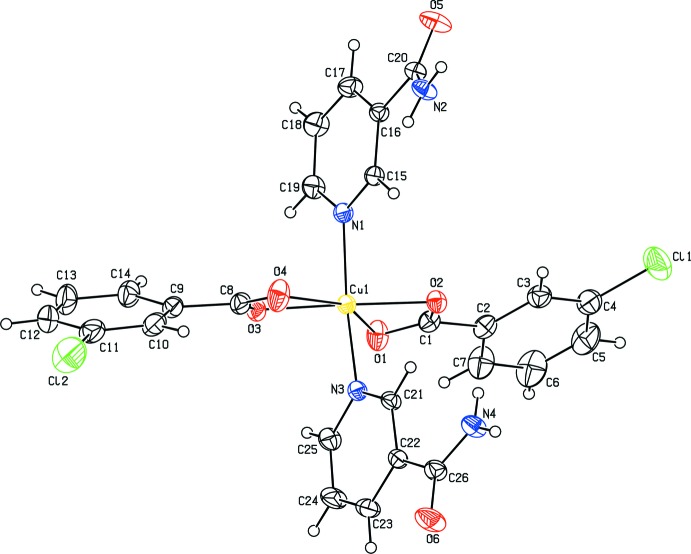
The molecular structure of the title compound with the atom–numbering scheme. Displacement ellipsoids are drawn at the 50% probability level. H atoms are presented as a small spheres of arbitrary radius.

## supplementary crystallographic information

### Comment

As a part of our ongoing investigation on transition metal complexes of nicotinamide (NA), one form of niacin (Krishnamachari, 1974), and/or the nicotinic acid derivative *N,N*–diethylnicotinamide, an important respiratory stimulant (Bigoli *et al.*, 1972), the title compound was synthesized and its crystal structure is reported herein.

In the monomeric title complex, I, the Cu^II^ ion is surrounded by two 3–chlorobenzoate (*CB*) and two (NA) ligands. The *CB* act as bidentate ligands, while the NA are monodentate ligands. The structures of similar complexes of Zn(II) and Cd(II) ions, [Zn_2_(C_10_H_14_N_2_O)_2_(C_7_H_5_O_3_)_4_]^.^2H_2_O, II, (Hökelek & Necefoğlu, 1996), [Zn(C_9_H_10_NO_2_)_2_(C_6_H_6_N_2_O)^.^2H_2_O], III, (Hökelek, Dal *et al.*, 2009) and [Cd(C_8_H_5_O_3_)_2_(C_6_H_6_N_2_O)_2_]^.^H_2_O, IV, (Hökelek, Yılmaz *et al.*, 2009) have also been determined.

In the title compound (Fig. 1), the Cu atom is displaced out of the least–squares planes of the carboxylate groups (O1/C1/O2) and (O3/C8/O4) by 0.1556 (2)Å and -0.0577 (2)Å, respectively. The dihedral angle between the planar carboxylate groups and the adjacent benzene rings *A* (C2—C7) and *B* (C9—C14) are 17.92 (12)° and 24.69 (16)°, respectively, while those between rings *A*, *B*, *C* (N1/C15—C19) and *D* (N3/C21—C25) are *A*/*B* = 52.20 (8)°, *A*/*C* = 85.61 (7)°, *A*/*D* = 84.86 (7)°, *B*/*C* = 71.49 (7)°, *B*/*D* = 69.95 (6)° and *C*/*D* = 1.56 (6)°. The two four–membered rings, (Cu1/O1/O2/C1) and (Cu1/O3/O4/C8), are oriented at a dihedral angle of 12.07 (7)°.

In I, the O1–Cu1–O2 and O3–Cu1–O4 angles are 59.76 (5)° and 55.08 (5)°, respectively. The corresponding O–*M*–O (where *M* is a metal) angles are 58.3 (3)° in II, 60.03 (6)° in III, 52.91 (4) and 53.96 (4)° in IV, 53.50 (14)° in [Cu_2_(C_8_H_5_O_3_)_4_(C_6_H_6_N_2_O)_4_], V, (Sertçelik *et al.*, 2013), 57.75 (2)° in [Cu(C_7_H_4_FO_2_)_2_(C_7_H_5_FO_2_)(C_6_H_6_N_2_O)_2_], VI, (Necefoğlu *et al.*, 2011), 58.3 (3)° in [Cu(C_7_H_5_O_2_)_2_(C_10_H_14_N_2_O)_2_], VII, (Hökelek *et al.*, 1996) and 55.2 (1)° in [Cu(*Asp*)_2_(*Py*)_2_], where *Asp* is acetylsalicylate and *Py* is pyridine, VIII, (Greenaway *et al.*, 1984).

In the crystal structure, intermolecular N—H···O and C—H···O hydrogen bonds (Table 1) link the molecules into a three–dimensional network, in which they may be effective in the stabilization of the structure. The π–π contact between the benzene rings, *Cg*2···*Cg*2^i^, where *Cg*2 is the centroid of the ring *B* (C9–C14) may further stabilize the structure, with Cg···Cg distance of 3.982 (2)Å. Symmetry code: (i) -*x*, 1-*y*, -*z*.

### Experimental

The title compound was prepared by the reaction of CuSO_4_^.^5H_2_O (1.25 g, 5 mmol) in H_2_O (50 ml) and NA (1.22 g, 10 mmol) in H_2_O (50 ml) with sodium 3–chlorobenzoate (1.79 g, 10 mmol) in H_2_O (100 ml). The mixture was filtered and set aside to crystallize at ambient temperature for one week, giving blue single crystals.

### Refinement

Atoms H2A, H2B, H4A and H4B (NH_2_ groups) were located in a difference Fourier map and refined isotropically. The remaining H atoms were positioned geometrically with C—H = 0.93Å for aromatic H atoms, and constrained to ride on their parent atoms, with *U*_iso_(H) = 1.2*U*_eq_(C).

### Crystal data

**Table:**

[Cu(C_7_H_4_ClO_2_)_2_(C_6_H_6_N_2_O)_2_]	*Z* = 2
*M**_r_* = 618.91	*F*(000) = 630
Triclinic, *P*1	*D*_x_ = 1.541 Mg m^−^^3^
Hall symbol: -P 1	Mo *K*α radiation, λ = 0.71073 Å
*a* = 9.6614 (2) Å	Cell parameters from 9977 reflections
*b* = 12.5429 (3) Å	θ = 2.2–28.3°
*c* = 12.8728 (3) Å	µ = 1.07 mm^−^^1^
α = 61.598 (2)°	*T* = 296 K
β = 87.386 (3)°	Block, blue
γ = 77.115 (3)°	0.35 × 0.20 × 0.15 mm
*V* = 1334.30 (6) Å^3^	

### Data collection

**Table:**

Bruker SMART BREEZE CCD diffractometer	5434 independent reflections
Radiation source: fine–focus sealed tube	4970 reflections with *I* > 2σ(*I*)
Graphite monochromator	*R*_int_ = 0.022
φ and ω scans	θ_max_ = 26.4°, θ_min_ = 1.8°
Absorption correction: multi-scan (*SADABS*; Bruker, 2012)	*h* = −12→12
*T*_min_ = 0.774, *T*_max_ = 0.852	*k* = −15→15
19053 measured reflections	*l* = −16→16

### Refinement

**Table:**

Refinement on *F*^2^	Primary atom site location: structure-invariant direct methods
Least-squares matrix: full	Secondary atom site location: difference Fourier map
*R*[*F*^2^ > 2σ(*F*^2^)] = 0.030	Hydrogen site location: inferred from neighbouring sites
*wR*(*F*^2^) = 0.083	H atoms treated by a mixture of independent and constrained refinement
*S* = 1.06	*w* = 1/[σ^2^(*F*_o_^2^) + (0.0431*P*)^2^ + 0.6228*P*] where *P* = (*F*_o_^2^ + 2*F*_c_^2^)/3
5434 reflections	(Δ/σ)_max_ = 0.001
368 parameters	Δρ_max_ = 0.43 e Å^−^^3^
117 restraints	Δρ_min_ = −0.43 e Å^−^^3^

### Special details

**Table:**

Geometry. All s.u.'s (except the s.u. in the dihedral angle between two l.s. planes) are estimated using the full covariance matrix. The cell s.u.'s are taken into account individually in the estimation of s.u.'s in distances, angles and torsion angles; correlations between s.u.'s in cell parameters are only used when they are defined by crystal symmetry. An approximate (isotropic) treatment of cell s.u.'s is used for estimating s.u.'s involving l.s. planes.
Refinement. Refinement of *F*^2^ against ALL reflections. The weighted *R*–factor *wR* and goodness of fit *S* are based on *F*^2^, conventional *R*–factors *R* are based on *F*, with *F* set to zero for negative *F*^2^. The threshold expression of *F*^2^ > σ(*F*^2^) is used only for calculating *R*–factors(gt) *etc*. and is not relevant to the choice of reflections for refinement. *R*–factors based on *F*^2^ are statistically about twice as large as those based on *F*, and *R*–factors based on ALL data will be even larger.

### Fractional atomic coordinates and isotropic or equivalent isotropic displacement parameters (Å^2^)

**Table:**

	*x*	*y*	*z*	*U*_iso_*/*U*_eq_	
Cu1	0.27888 (2)	1.000999 (18)	0.199774 (17)	0.02922 (8)	
Cl1	0.80542 (9)	0.43210 (7)	0.58781 (9)	0.0953 (3)	
Cl2	−0.34732 (8)	1.57612 (7)	−0.04862 (10)	0.1008 (3)	
O1	0.38881 (17)	0.84400 (13)	0.15270 (12)	0.0491 (4)	
O2	0.39807 (13)	0.83886 (11)	0.32503 (11)	0.0345 (3)	
O3	0.18430 (14)	1.14369 (12)	0.05257 (11)	0.0375 (3)	
O4	0.0977 (2)	1.19856 (14)	0.18577 (12)	0.0570 (4)	
O5	0.76794 (14)	1.14536 (16)	0.40191 (15)	0.0545 (4)	
O6	−0.25440 (15)	0.82581 (19)	0.51564 (15)	0.0641 (5)	
N1	0.43976 (16)	1.08071 (13)	0.18924 (12)	0.0318 (3)	
N2	0.54419 (18)	1.16359 (17)	0.45497 (15)	0.0398 (4)	
H2A	0.565 (2)	1.176 (2)	0.507 (2)	0.040 (6)*	
H2B	0.458 (3)	1.172 (2)	0.440 (2)	0.050 (6)*	
N3	0.10420 (15)	0.93169 (13)	0.24499 (12)	0.0315 (3)	
N4	−0.04948 (19)	0.83277 (19)	0.58345 (16)	0.0435 (4)	
H4A	0.034 (3)	0.841 (2)	0.574 (2)	0.048 (6)*	
H4B	−0.079 (3)	0.818 (2)	0.648 (2)	0.058 (7)*	
C1	0.4330 (2)	0.78886 (17)	0.25815 (16)	0.0354 (4)	
C2	0.5297 (2)	0.66138 (17)	0.31005 (18)	0.0411 (4)	
C3	0.6115 (2)	0.61181 (18)	0.4143 (2)	0.0448 (5)	
H3	0.6067	0.6566	0.4553	0.054*	
C4	0.7010 (2)	0.4944 (2)	0.4575 (2)	0.0551 (6)	
C5	0.7073 (3)	0.4258 (2)	0.3993 (3)	0.0701 (7)	
H5	0.7664	0.3465	0.4298	0.084*	
C6	0.6252 (4)	0.4758 (2)	0.2957 (3)	0.0791 (9)	
H6	0.6283	0.4298	0.2560	0.095*	
C7	0.5381 (3)	0.5933 (2)	0.2499 (2)	0.0640 (7)	
H7	0.4848	0.6272	0.1785	0.077*	
C8	0.1053 (2)	1.21991 (17)	0.08210 (16)	0.0377 (4)	
C9	0.0182 (2)	1.33707 (17)	−0.01605 (17)	0.0391 (4)	
C10	−0.1053 (2)	1.39805 (18)	0.0098 (2)	0.0487 (5)	
H10	−0.1309	1.3691	0.0875	0.058*	
C11	−0.1900 (2)	1.5022 (2)	−0.0812 (2)	0.0582 (6)	
C12	−0.1515 (3)	1.5492 (2)	−0.1967 (2)	0.0668 (7)	
H12	−0.2098	1.6192	−0.2573	0.080*	
C13	−0.0263 (3)	1.4911 (2)	−0.2205 (2)	0.0640 (6)	
H13	0.0021	1.5236	−0.2975	0.077*	
C14	0.0587 (3)	1.3845 (2)	−0.13122 (18)	0.0498 (5)	
H14	0.1426	1.3448	−0.1486	0.060*	
C15	0.47759 (18)	1.09464 (16)	0.28005 (14)	0.0307 (3)	
H15	0.4211	1.0749	0.3442	0.037*	
C16	0.59731 (17)	1.13714 (15)	0.28251 (15)	0.0297 (3)	
C17	0.6804 (2)	1.16596 (19)	0.18655 (18)	0.0412 (4)	
H17	0.7637	1.1914	0.1866	0.049*	
C18	0.6388 (2)	1.1566 (2)	0.09122 (18)	0.0474 (5)	
H18	0.6915	1.1789	0.0248	0.057*	
C19	0.5179 (2)	1.11393 (19)	0.09505 (16)	0.0405 (4)	
H19	0.4899	1.1080	0.0302	0.049*	
C20	0.64242 (18)	1.14888 (16)	0.38547 (16)	0.0334 (4)	
C21	0.05121 (18)	0.90891 (16)	0.34942 (15)	0.0308 (3)	
H21	0.0993	0.9225	0.4014	0.037*	
C22	−0.07235 (18)	0.86597 (16)	0.38352 (15)	0.0319 (4)	
C23	−0.1438 (2)	0.8479 (2)	0.30439 (18)	0.0444 (5)	
H23	−0.2282	0.8210	0.3234	0.053*	
C24	−0.0884 (2)	0.8701 (2)	0.19695 (19)	0.0494 (5)	
H24	−0.1344	0.8574	0.1432	0.059*	
C25	0.0353 (2)	0.91112 (19)	0.17077 (17)	0.0408 (4)	
H25	0.0728	0.9252	0.0987	0.049*	
C26	−0.13259 (19)	0.84043 (18)	0.50037 (17)	0.0380 (4)	

### Atomic displacement parameters (Å^2^)

**Table:**

	*U*^11^	*U*^22^	*U*^33^	*U*^12^	*U*^13^	*U*^23^
Cu1	0.03384 (13)	0.03311 (12)	0.02251 (11)	−0.01037 (9)	0.00167 (8)	−0.01354 (9)
Cl1	0.0746 (5)	0.0701 (4)	0.1137 (7)	0.0100 (4)	−0.0438 (5)	−0.0290 (4)
Cl2	0.0536 (4)	0.0683 (4)	0.1427 (8)	0.0039 (3)	0.0142 (4)	−0.0287 (5)
O1	0.0689 (10)	0.0471 (8)	0.0279 (7)	−0.0023 (7)	0.0038 (6)	−0.0200 (6)
O2	0.0412 (7)	0.0358 (6)	0.0285 (6)	−0.0055 (5)	0.0001 (5)	−0.0180 (5)
O3	0.0451 (7)	0.0373 (6)	0.0279 (6)	−0.0085 (6)	−0.0017 (5)	−0.0138 (5)
O4	0.0919 (12)	0.0461 (8)	0.0290 (7)	−0.0090 (8)	0.0040 (7)	−0.0175 (6)
O5	0.0295 (7)	0.0882 (11)	0.0697 (10)	−0.0180 (7)	0.0031 (7)	−0.0548 (9)
O6	0.0336 (8)	0.1099 (14)	0.0543 (10)	−0.0324 (8)	0.0127 (7)	−0.0376 (10)
N1	0.0362 (7)	0.0352 (7)	0.0256 (7)	−0.0118 (6)	0.0024 (6)	−0.0144 (6)
N2	0.0334 (9)	0.0622 (11)	0.0368 (9)	−0.0152 (7)	0.0031 (7)	−0.0322 (8)
N3	0.0344 (7)	0.0346 (7)	0.0266 (7)	−0.0093 (6)	0.0006 (6)	−0.0148 (6)
N4	0.0315 (9)	0.0711 (12)	0.0331 (9)	−0.0195 (8)	0.0094 (7)	−0.0262 (8)
C1	0.0398 (9)	0.0360 (9)	0.0327 (9)	−0.0111 (7)	0.0080 (7)	−0.0177 (7)
C2	0.0451 (11)	0.0366 (9)	0.0419 (10)	−0.0110 (8)	0.0123 (8)	−0.0192 (8)
C3	0.0408 (10)	0.0402 (10)	0.0536 (12)	−0.0091 (8)	0.0040 (9)	−0.0226 (9)
C4	0.0400 (11)	0.0442 (11)	0.0685 (15)	−0.0062 (9)	0.0014 (10)	−0.0181 (11)
C5	0.0697 (16)	0.0405 (12)	0.088 (2)	0.0011 (11)	0.0127 (14)	−0.0276 (13)
C6	0.114 (2)	0.0505 (14)	0.0786 (19)	−0.0030 (15)	0.0115 (17)	−0.0426 (14)
C7	0.0931 (19)	0.0480 (12)	0.0525 (14)	−0.0053 (12)	0.0044 (13)	−0.0300 (11)
C8	0.0476 (10)	0.0350 (9)	0.0313 (9)	−0.0130 (8)	0.0014 (8)	−0.0148 (7)
C9	0.0482 (11)	0.0327 (9)	0.0353 (10)	−0.0133 (8)	0.0000 (8)	−0.0134 (8)
C10	0.0497 (12)	0.0373 (10)	0.0529 (12)	−0.0149 (9)	0.0078 (9)	−0.0148 (9)
C11	0.0430 (12)	0.0389 (11)	0.0801 (17)	−0.0089 (9)	−0.0009 (11)	−0.0183 (11)
C12	0.0701 (16)	0.0426 (12)	0.0636 (16)	−0.0101 (11)	−0.0198 (13)	−0.0054 (11)
C13	0.0832 (18)	0.0534 (13)	0.0370 (12)	−0.0156 (12)	−0.0041 (11)	−0.0065 (10)
C14	0.0611 (13)	0.0457 (11)	0.0361 (10)	−0.0123 (10)	0.0024 (9)	−0.0144 (9)
C15	0.0330 (8)	0.0365 (8)	0.0243 (8)	−0.0123 (7)	0.0053 (6)	−0.0143 (7)
C16	0.0282 (8)	0.0321 (8)	0.0296 (8)	−0.0081 (6)	0.0031 (6)	−0.0150 (7)
C17	0.0364 (10)	0.0513 (11)	0.0419 (10)	−0.0188 (8)	0.0117 (8)	−0.0239 (9)
C18	0.0488 (11)	0.0652 (13)	0.0333 (10)	−0.0234 (10)	0.0183 (8)	−0.0245 (10)
C19	0.0464 (11)	0.0525 (11)	0.0277 (9)	−0.0152 (9)	0.0073 (8)	−0.0219 (8)
C20	0.0296 (8)	0.0374 (9)	0.0374 (9)	−0.0096 (7)	0.0001 (7)	−0.0203 (8)
C21	0.0312 (8)	0.0375 (9)	0.0276 (8)	−0.0104 (7)	0.0000 (6)	−0.0174 (7)
C22	0.0286 (8)	0.0351 (8)	0.0315 (9)	−0.0074 (7)	−0.0009 (7)	−0.0151 (7)
C23	0.0392 (10)	0.0553 (12)	0.0450 (11)	−0.0207 (9)	−0.0010 (8)	−0.0244 (10)
C24	0.0547 (12)	0.0657 (13)	0.0436 (11)	−0.0237 (11)	−0.0028 (9)	−0.0339 (10)
C25	0.0492 (11)	0.0500 (11)	0.0302 (9)	−0.0150 (9)	0.0018 (8)	−0.0231 (8)
C26	0.0279 (9)	0.0484 (10)	0.0370 (10)	−0.0114 (8)	0.0050 (7)	−0.0188 (8)

### Geometric parameters (Å, º)

**Table:**

Cu1—O1	2.3487 (14)	C6—H6	0.9300
Cu1—O2	2.0168 (12)	C7—C6	1.376 (4)
Cu1—O3	1.9574 (12)	C7—H7	0.9300
Cu1—O4	2.6280 (12)	C8—C9	1.498 (3)
Cu1—N1	1.9947 (14)	C9—C10	1.385 (3)
Cu1—N3	2.0065 (14)	C9—C14	1.384 (3)
Cu1—C1	2.5090 (18)	C10—C11	1.379 (3)
Cl1—C4	1.731 (3)	C10—H10	0.9300
Cl2—C11	1.740 (3)	C11—C12	1.382 (4)
O1—C1	1.237 (2)	C12—H12	0.9300
O2—C1	1.281 (2)	C13—C12	1.369 (4)
O3—C8	1.279 (2)	C13—H13	0.9300
O4—C8	1.232 (2)	C14—C13	1.385 (3)
O5—C20	1.228 (2)	C14—H14	0.9300
O6—C26	1.224 (2)	C15—H15	0.9300
N1—C15	1.339 (2)	C16—C15	1.385 (2)
N1—C19	1.338 (2)	C16—C17	1.384 (2)
N2—C20	1.318 (2)	C16—C20	1.495 (2)
N2—H2A	0.79 (2)	C17—C18	1.373 (3)
N2—H2B	0.84 (3)	C17—H17	0.9300
N3—C21	1.337 (2)	C18—H18	0.9300
N3—C25	1.338 (2)	C19—C18	1.379 (3)
N4—C26	1.321 (2)	C19—H19	0.9300
N4—H4A	0.83 (3)	C21—C22	1.386 (2)
N4—H4B	0.81 (3)	C21—H21	0.9300
C1—C2	1.500 (3)	C22—C23	1.385 (3)
C2—C3	1.377 (3)	C22—C26	1.500 (3)
C2—C7	1.388 (3)	C23—C24	1.382 (3)
C3—C4	1.387 (3)	C23—H23	0.9300
C3—H3	0.9300	C24—H24	0.9300
C4—C5	1.376 (4)	C25—C24	1.371 (3)
C5—C6	1.371 (4)	C25—H25	0.9300
C5—H5	0.9300		
			
O1—Cu1—C1	29.28 (5)	O4—C8—O3	122.57 (17)
O2—Cu1—O1	59.76 (5)	O4—C8—C9	120.80 (18)
O2—Cu1—C1	30.48 (5)	C10—C9—C8	118.89 (18)
O3—Cu1—O1	106.81 (5)	C14—C9—C8	121.26 (19)
O3—Cu1—O2	166.26 (5)	C14—C9—C10	119.85 (19)
O3—Cu1—O4	55.08 (5)	C9—C10—H10	120.4
O3—Cu1—N1	91.50 (6)	C11—C10—C9	119.2 (2)
O3—Cu1—N3	93.10 (6)	C11—C10—H10	120.4
O3—Cu1—C1	136.00 (6)	C10—C11—Cl2	119.0 (2)
N1—Cu1—O1	100.98 (6)	C10—C11—C12	121.3 (2)
N1—Cu1—O2	88.65 (5)	C12—C11—Cl2	119.71 (19)
N1—Cu1—N3	164.90 (6)	C11—C12—H12	120.5
N1—Cu1—C1	95.33 (6)	C13—C12—C11	119.0 (2)
N3—Cu1—O1	91.43 (6)	C13—C12—H12	120.5
N3—Cu1—O2	90.26 (5)	C12—C13—C14	120.7 (2)
N3—Cu1—C1	91.32 (6)	C12—C13—H13	119.6
C1—O1—Cu1	82.56 (11)	C14—C13—H13	119.6
C1—O2—Cu1	96.52 (11)	C9—C14—C13	119.8 (2)
C8—O3—Cu1	106.27 (11)	C9—C14—H14	120.1
C15—N1—Cu1	120.40 (11)	C13—C14—H14	120.1
C19—N1—Cu1	120.98 (12)	N1—C15—C16	122.74 (16)
C19—N1—C15	118.47 (15)	N1—C15—H15	118.6
C20—N2—H2A	119.4 (16)	C16—C15—H15	118.6
C20—N2—H2B	121.7 (17)	C15—C16—C20	122.86 (15)
H2A—N2—H2B	118 (2)	C17—C16—C15	117.97 (16)
C21—N3—Cu1	120.20 (11)	C17—C16—C20	119.14 (16)
C21—N3—C25	118.42 (15)	C16—C17—H17	120.3
C25—N3—Cu1	121.34 (13)	C18—C17—C16	119.43 (17)
C26—N4—H4A	122.8 (16)	C18—C17—H17	120.3
C26—N4—H4B	119.5 (18)	C17—C18—C19	119.23 (17)
H4A—N4—H4B	118 (2)	C17—C18—H18	120.4
O1—C1—Cu1	68.16 (10)	C19—C18—H18	120.4
O1—C1—O2	121.15 (17)	N1—C19—C18	122.04 (17)
O1—C1—C2	120.21 (17)	N1—C19—H19	119.0
O2—C1—Cu1	53.00 (9)	C18—C19—H19	119.0
O2—C1—C2	118.64 (16)	O5—C20—N2	122.54 (17)
C2—C1—Cu1	171.55 (14)	O5—C20—C16	119.44 (16)
C3—C2—C1	121.54 (18)	N2—C20—C16	118.02 (15)
C3—C2—C7	119.6 (2)	N3—C21—C22	122.83 (15)
C7—C2—C1	118.8 (2)	N3—C21—H21	118.6
C2—C3—C4	119.3 (2)	C22—C21—H21	118.6
C2—C3—H3	120.4	C21—C22—C26	123.15 (15)
C4—C3—H3	120.4	C23—C22—C21	117.85 (17)
C3—C4—Cl1	119.5 (2)	C23—C22—C26	118.99 (16)
C5—C4—Cl1	119.32 (19)	C22—C23—H23	120.3
C5—C4—C3	121.1 (2)	C24—C23—C22	119.42 (18)
C4—C5—H5	120.4	C24—C23—H23	120.3
C6—C5—C4	119.1 (2)	C23—C24—H24	120.5
C6—C5—H5	120.4	C25—C24—C23	118.95 (17)
C5—C6—C7	120.6 (3)	C25—C24—H24	120.5
C5—C6—H6	119.7	N3—C25—C24	122.50 (18)
C7—C6—H6	119.7	N3—C25—H25	118.8
C2—C7—H7	119.9	C24—C25—H25	118.8
C6—C7—C2	120.2 (3)	O6—C26—N4	122.87 (19)
C6—C7—H7	119.9	O6—C26—C22	119.36 (17)
O3—C8—C9	116.61 (16)	N4—C26—C22	117.76 (16)
			
O2—Cu1—O1—C1	−0.68 (11)	Cu1—N3—C21—C22	177.25 (13)
O3—Cu1—O1—C1	176.18 (11)	C25—N3—C21—C22	−0.5 (3)
N1—Cu1—O1—C1	81.22 (12)	Cu1—N3—C25—C24	−176.47 (16)
N3—Cu1—O1—C1	−90.14 (12)	C21—N3—C25—C24	1.3 (3)
O1—Cu1—O2—C1	0.66 (10)	O1—C1—C2—C3	161.79 (19)
O3—Cu1—O2—C1	−12.1 (3)	O1—C1—C2—C7	−17.1 (3)
N1—Cu1—O2—C1	−102.89 (11)	O2—C1—C2—C3	−18.0 (3)
N3—Cu1—O2—C1	92.17 (11)	O2—C1—C2—C7	163.1 (2)
O1—Cu1—O3—C8	169.20 (11)	C1—C2—C3—C4	−178.91 (18)
O2—Cu1—O3—C8	−179.33 (19)	C7—C2—C3—C4	0.0 (3)
N1—Cu1—O3—C8	−88.86 (12)	C1—C2—C7—C6	−179.5 (2)
N3—Cu1—O3—C8	76.76 (12)	C3—C2—C7—C6	1.5 (4)
C1—Cu1—O3—C8	171.88 (11)	C2—C3—C4—Cl1	179.33 (16)
O1—Cu1—N1—C15	−126.37 (13)	C2—C3—C4—C5	−1.3 (3)
O1—Cu1—N1—C19	49.23 (15)	Cl1—C4—C5—C6	−179.5 (2)
O2—Cu1—N1—C15	−67.55 (14)	C3—C4—C5—C6	1.1 (4)
O2—Cu1—N1—C19	108.05 (15)	C4—C5—C6—C7	0.4 (5)
O3—Cu1—N1—C15	126.19 (14)	C2—C7—C6—C5	−1.7 (5)
O3—Cu1—N1—C19	−58.21 (15)	O3—C8—C9—C10	155.12 (18)
N3—Cu1—N1—C15	18.4 (3)	O3—C8—C9—C14	−24.5 (3)
N3—Cu1—N1—C19	−165.97 (19)	O4—C8—C9—C10	−23.5 (3)
C1—Cu1—N1—C15	−97.33 (14)	O4—C8—C9—C14	156.8 (2)
C1—Cu1—N1—C19	78.27 (15)	C8—C9—C10—C11	−176.52 (18)
O1—Cu1—N3—C21	128.70 (13)	C14—C9—C10—C11	3.1 (3)
O1—Cu1—N3—C25	−53.57 (15)	C8—C9—C14—C13	178.2 (2)
O2—Cu1—N3—C21	68.94 (13)	C10—C9—C14—C13	−1.4 (3)
O2—Cu1—N3—C25	−113.33 (15)	C9—C10—C11—Cl2	178.44 (17)
O3—Cu1—N3—C21	−124.39 (13)	C9—C10—C11—C12	−2.3 (3)
O3—Cu1—N3—C25	53.34 (15)	Cl2—C11—C12—C13	178.9 (2)
N1—Cu1—N3—C21	−16.8 (3)	C10—C11—C12—C13	−0.4 (4)
N1—Cu1—N3—C25	160.9 (2)	C14—C13—C12—C11	2.1 (4)
C1—Cu1—N3—C21	99.41 (13)	C9—C14—C13—C12	−1.2 (4)
C1—Cu1—N3—C25	−82.86 (15)	C17—C16—C15—N1	−0.1 (3)
O1—Cu1—C1—O2	−178.84 (18)	C20—C16—C15—N1	−177.97 (16)
O2—Cu1—C1—O1	178.84 (18)	C15—C16—C17—C18	2.7 (3)
O3—Cu1—C1—O1	−5.26 (15)	C20—C16—C17—C18	−179.32 (18)
O3—Cu1—C1—O2	175.90 (9)	C15—C16—C20—O5	155.40 (18)
N1—Cu1—C1—O1	−102.99 (12)	C15—C16—C20—N2	−25.1 (3)
N1—Cu1—C1—O2	78.17 (11)	C17—C16—C20—O5	−22.5 (3)
N3—Cu1—C1—O1	90.58 (12)	C17—C16—C20—N2	157.06 (18)
N3—Cu1—C1—O2	−88.26 (11)	C16—C17—C18—C19	−2.6 (3)
Cu1—O1—C1—O2	1.09 (17)	N1—C19—C18—C17	−0.2 (3)
Cu1—O1—C1—C2	−178.70 (16)	N3—C21—C22—C23	−0.8 (3)
Cu1—O2—C1—O1	−1.3 (2)	N3—C21—C22—C26	−179.89 (16)
Cu1—O2—C1—C2	178.53 (14)	C21—C22—C23—C24	1.5 (3)
Cu1—O3—C8—O4	−0.1 (2)	C26—C22—C23—C24	−179.42 (19)
Cu1—O3—C8—C9	−178.74 (13)	C21—C22—C26—O6	166.2 (2)
Cu1—N1—C15—C16	173.02 (13)	C21—C22—C26—N4	−14.9 (3)
C19—N1—C15—C16	−2.7 (3)	C23—C22—C26—O6	−12.9 (3)
Cu1—N1—C19—C18	−172.84 (16)	C23—C22—C26—N4	166.07 (19)
C15—N1—C19—C18	2.8 (3)		

### Hydrogen-bond geometry (Å, º)

**Table:**

*D*—H···*A*	*D*—H	H···*A*	*D*···*A*	*D*—H···*A*
N2—H2*A*···O2^i^	0.80 (2)	2.12 (2)	2.896 (2)	164 (2)
N2—H2*B*···O6^ii^	0.84 (3)	2.02 (3)	2.790 (2)	153 (2)
N4—H4*A*···O5^i^	0.83 (3)	2.01 (3)	2.817 (2)	164 (2)
N4—H4*B*···O4^ii^	0.81 (2)	2.05 (2)	2.836 (2)	162 (3)
C19—H19···O1^iii^	0.93	2.45	3.100 (2)	127
C21—H21···O5^i^	0.93	2.56	3.416 (2)	154
C24—H24···O3^iv^	0.93	2.59	3.475 (3)	158

Symmetry codes: (i) −*x*+1, −*y*+2, −*z*+1; (ii) −*x*, −*y*+2, −*z*+1; (iii) −*x*+1, −*y*+2, −*z*; (iv) −*x*, −*y*+2, −*z*.
